# Identification of the PLK2-Dependent Phosphopeptidome by Quantitative Proteomics

**DOI:** 10.1371/journal.pone.0111018

**Published:** 2014-10-22

**Authors:** Cinzia Franchin, Luca Cesaro, Lorenzo A. Pinna, Giorgio Arrigoni, Mauro Salvi

**Affiliations:** 1 Department of Biomedical Sciences, University of Padova, Padova, Italy; 2 Proteomics Center of Padova University, Padova, Italy; 3 CNR Institute of Neurosciences, Padova, Italy; National Cancer Institute, NIH, United States of America

## Abstract

Polo-like kinase 2 (PLK2) has been recently recognized as the major enzyme responsible for phosphorylation of α-synuclein at S129 *in vitro* and *in vivo*, suggesting that this kinase may play a key role in the pathogenesis of Parkinson's disease and other synucleinopathies. Moreover PLK2 seems to be implicated in cell division, oncogenesis, and synaptic regulation of the brain. However little is known about the phosphoproteome generated by PLK2 and, consequently the overall impact of PLK2 on cellular signaling. To fill this gap we exploited an approach based on *in vitro* kinase assay and quantitative phosphoproteomics. A proteome-derived peptide library obtained by digestion of undifferentiated human neuroblastoma cell line was exhaustively dephosphorylated by lambda phosphatase followed by incubation with or without PLK2 recombinant kinase. Stable isotope labeling based quantitative phosphoproteomics was applied to identify the phosphosites generated by PLK2. A total of 98 unique PLK2-dependent phosphosites from 89 proteins were identified by LC-MS/MS. Analysis of the primary structure of the identified phosphosites allowed the detailed definition of the kinase specificity and the compilation of a list of potential PLK2 targets among those retrieved in PhosphositePlus, a curated database of in cell*/vivo* phosphorylation sites.

## Introduction

The Polo like-kinase 2 (PLK2) is a serine/threonine kinase belonging to the POLO like kinase family playing a role in cell cycle progression, mitosis, cytokinesis, and DNA damage response. In mammals, five members of this family have been described: the best characterized PLK1, the closely related PLK3 and PLK2, a distant member PLK4, and PLK5, a protein that lacks the kinase domain in humans. The members of this family share the same domain topology, consisting of a conserved N-terminal kinase domain and one or two POLO box domains at the C-terminus [1], [2], [3]. PLK2 was initially named Serum inducible kinase (Snk) having been classified as an early response gene as its expression increases following stimulation by growth factors. PLK2 is involved in cell cycle regulation, is required for centriole duplication in mammalian cells [4], regulates mitotic spindle in the mammary gland [5], and is a direct transcriptional target of p53 activating G2-M checkpoint, which prevents mitotic catastrophe following spindle damage [6].

While PLK1 has been pre-clinically validated as a cancer target and is generally overexpressed in different forms of human tumors [7], PLK2 has been initially described as a tumor suppressor gene [3]. However recent works disclose a more complex scenario where also PLK2 inhibition has been suggested as a promising therapeutic strategy against some type of tumors. In this regard PLK2 can bind and phosphorylate the mutant p53, inducing an oncogenic feedback loop in cancer cells [8], or may promote Mcl-1 stabilization, thus providing resistance to cell death induced by TRAIL in Cholangiocarcinoma [9].

Moreover, PLK2 is required for the regulation of the homeostatic synaptic plasticity in the brain: PLK2 acts on Ras and Rap signaling by phosphorylating four Ras and Rap regulators [10]. Recently PLK2 took the center of the stage after being identified as the major kinase responsible for the phosphorylation of Ser-129 of α-synuclein both *in vitro* and *in vivo* [11], [12], [13], [14]. α-Synuclein is constitutively phosphorylated at low levels in normal brain and an accumulation of α-synuclein pS129 in Lewy bodies is observed in Parkinson disease and other synucleinopathies. Although the pathophysiology of the Ser-129 phosphorylation in Parkinson's disease is not completely understood and it has not been clarified whether this phosphorylation is protective or harmful for neurons, PLK2 is considered a very promising target for Parkinson disease treatment [15], [16], [17].

Despite the fact that the involvement of PLK2 in different biological processes is emerging, the precise functions of this kinase remain elusive as, with few exceptions, its main cellular targets are unknown. Indeed, the PLK2 substrates identified so far are just a dozen or so and the phosphoresidues are often not characterized.

We have here exploited a strategy based on *in vitro* kinase phosphorylation of proteome-derived peptide libraries combined with a mass spectrometry-based quantitative proteomic approach to identify the PLK2-dependent phosphopeptidome. A similar approach was successfully applied by Zou's group to identify putative substrates of the protein kinase CK2 [18]. Our analysis allowed for the detailed definition of the PLK2 kinase specificity and the compilation of a list of its potential targets to gain a deeper understanding of the involvement of this kinase in signal transduction pathways.

## Materials and Methods

### Materials

Recombinant human Dopa decarboxylase, Annexin A2 and Prostaglandin E Synthase 3 were purchased from ProSpec (Tany TechnoGene Ltd.). All chemicals and solvents were of MS-grade.

### c-DNA constructs and production of recombinant proteins

Plasmids encoding human GST-HDGF [19] and human PLK2-PGEX4TI [20] were previously described. GST-PLK2 T210D constitutively active mutant and GST-HDGF T225A were produced by PCR site-directed mutagenesis and mutations were confirmed by sequencing analysis.

Recombinant GST-HDGF, GST-CK2, and GST-PLK2 T210D, have been expressed in *E. coli* BL-21 pLysS and purified as described in [19] and [20], respectively.

### Cell culture

Human neuroblastoma SK-N-BE cells [21] were maintained in 5% CO_2_ in DMEM supplemented with 10% FBS, 2 mM l-glutamine, 100 U/ml penicillin and 100 mM streptomycin, in an atmosphere containing 5% CO_2_.

### Cell lysate dephosphorylation and in vitro assay

Undifferentiated cells were detached, centrifuged, extensively washed with PBS and lysed by the addition of ice-cold buffer containing 8 M urea in 25 mM Hepes (pH 8.0), protease inhibitor cocktail Complete (Roche) and ultrasonicated in an ice-bath. After 40 min, the lysate was centrifuged 15 min at 10000 × g at 4°C. The supernatant was collected and protein concentration was measured by BCA method.

Extracted proteins (2 mg) were reduced with 20 mM dithiothreitol for 1 h at 56°C and alkylated with 40 mM iodoacetamide for 45 min at room temperature in the dark. The sample was diluted 8 times with 25 mM Hepes pH 8.0 to reach a concentration of urea compatible with trypsin activity. Sequencing grade modified trypsin (45 µg) (Promega) was added to the sample and the protein mixture was digested at 37°C overnight.

Tryptic peptides were acidified with formic acid and desalted on SepPak Vac 1cc C18 Cartridges (Waters) following the manufacturer's instructions. Eluted peptides were dried under vacuum and then dissolved in 0.5 mL of dephosphorylation reaction buffer containing 50 mM Hepes pH 7.5, 2 mM MnCl_2_, 0.1 mM EGTA, 5 mM DTT and 0.01% BRIJ35. Dephosphorylation of peptides was carried out by adding 2000 U of lambda phosphatase (Santa Crutz). After 7 h at 37°C, other 2000 U of lambda phosphatase was added. This second dephosphorylation reaction was carried out overnight at 37°C. Finally the solution was heated at 95°C for 15 min to inactivate the phosphatase and subjected to *in vitro* phosphorylation. PLK2 phosphorylation conditions are described in [22]. Briefly, the sample was divided into two identical aliquots of 250 µl and each of them was diluted to 500 µL with a solution 2× containing 20 mM MgCl_2_, 10 mM DTT, and 200 µM ATP. One of the aliquots was supplemented with PLK2-GST T210D (1 µg) and both aliquots were incubated for 2h at 30°C. After incubation the samples were frozen and dried.

### Dimethyl labeling and phosphopeptides enrichment

**Figure 1:**
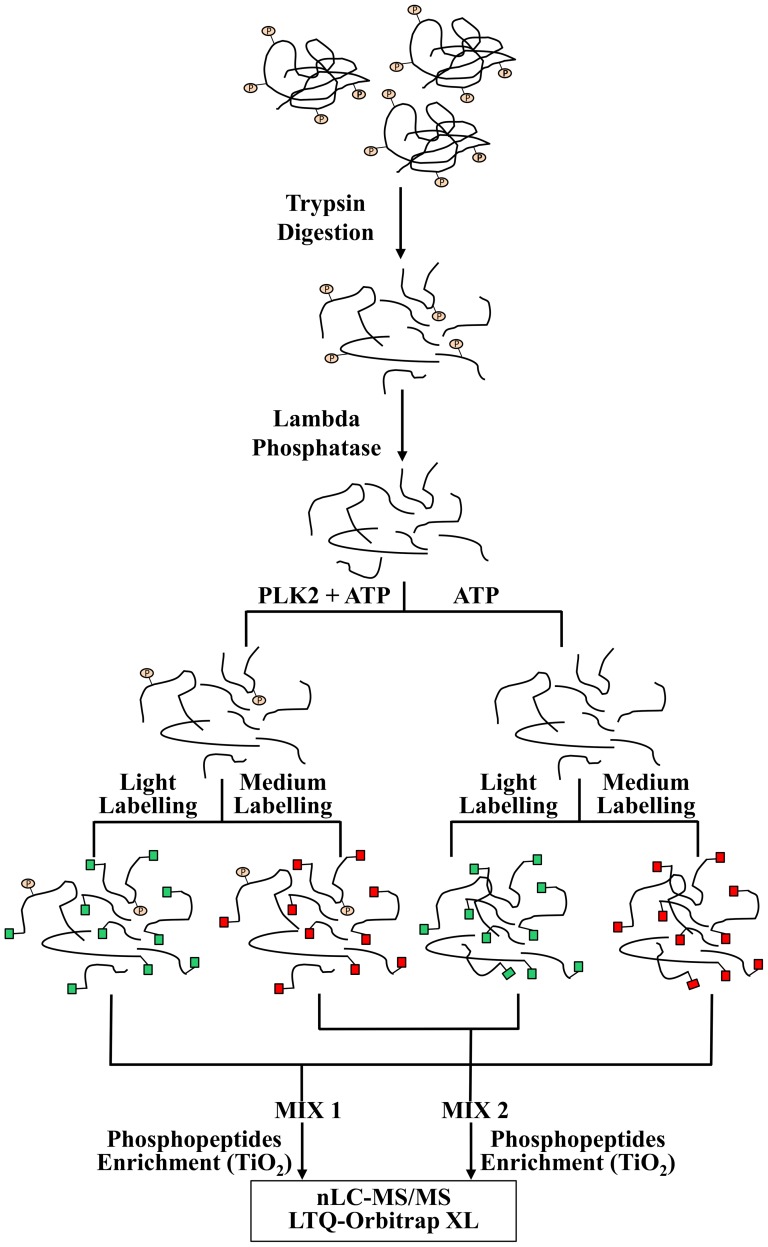
Workflow for PLK2 peptide substrate identification.

Samples were labeled according to the dimethyl labeling method described in [23] and following the scheme reported in Figure 1. 400 µg of each peptide solution (control sample and PLK2 phosphorylated sample) was diluted to 500 µl of 5% formic acid. Each sample was then divided into two identical aliquots of 250 µl to perform a “forward” and a “reverse” experiment. Two isotopic forms of formaldehyde were used: the “light” form (CH_2_O) and the “medium” form (CD_2_O). Labeling was performed on-column using SepPak Vac 1cc C18 Cartridges, as described in [23]. Samples were mixed in a 1∶1 ratio as described in Figure 1 and dried under vacuum.

Peptides from each of the two samples were dissolved in 100 µl of 80% acetonitrile, 6% of trifluoroacetic acid and phosphopeptides enrichment was performed using home-made micro columns packed with 400 µg of TiO_2_ (Titansphere) as described in [20]. Eluted peptides were acidified with formic acid, dried under vacuum, and samples were finally dissolved in 45 µl of 3% acetonitrile 0.1% formic acid just prior to LC-MS/MS analysis.

### Mass Spectrometry analysis

Mass spectrometry analyses were performed on an LTQ-Orbitrap XL mass spectrometer (Thermo Fisher Scientific) coupled with an on-line nano-HPLC Ultimate 3000 (Dionex – Thermo Fisher Scientific). Peptides were loaded onto a Trap column (300 mm I.D., 300 Å, C18, 3 mm; SGE Analytical Science) using a flow rate of 8 µL/min of 0.1% formic acid (solvent A), transferred into a homemade pico-frit column packed with C18 material (Aeris Peptide 3.6 µm XB-C18, Phenomenex), and separated using a linear gradient of acetonitrile/0.1% formic acid (solvent B) from 3% to 50% in 90 minutes at a flow rate of 250 µL/min. Ion source capillary temperature was set at 200°C, and spray voltage at 1.5 kV. To increase the number of identified phosphopeptides, each sample was analyzed three times with the same chromatographic conditions but using different fragmentation methods as described in [24].

### Data analysis

For each of the two final samples, MS/MS data derived from the different analyses were analyzed with a MudPIT protocol using Proteome Discoverer 1.4 software (Thermo Fisher Scientific) interfaced to a Mascot server (version 2.2.4, Matrix Science, London, UK). Searches were performed against the Uniprot Human protein database (version 2014.01.22, 88479 sequences). Enzyme specificity was set to trypsin and a maximum of two missed cleavages were allowed. The precursor and fragment mass tolerances were set to 10 ppm and 0.6 Da respectively. Light-marked dimethylation (+28.0313 Da) and medium-marked dimethylation (+32.0564 Da) were selected as variable modifications at N-terminus and lysine residues. Phosphorylation of serine, threonine, and tyrosine were also inserted as variable modifications, while carbamidomethylation of cysteines was set as static modification. The search was done also against a randomized database and the confidence level of all the identified peptides was assessed using the Percolator algorithm, and only peptides with a q-value <0.05 were considered as correctly identified. For quantification, all data were reported as “PLK2-treated” over control, with a maximum ratio of 100.

### *In vitro* phosphorylation

*In vitro* PLK2 phosphorylation assays were performed as described in [22]. Briefly, recombinant proteins were incubated at the indicated concentrations in a radioactive mixture consisting in 50 mM Tris (pH 7.5), 100 µM ATP ([γ-^33^P]ATP ∼ 2000 cpm/pmol), 10 mM MgCl_2_, and 5 mM DTT, in absence (control) or with GST-PLK2 T210D (20 ng) at 37°C for 10 min. For CK2 *in vitro* phosphorylation assay, protein substrate was incubated in the same radioactive mixture, without DTT and in presence of the GST-CK2 kinase (20 ng). The reaction was stopped with the addition of 2× Laemmli sample buffer and samples were subjected to SDS-PAGE. Gels were stained with colloidal coomassie, dried, exposed overnight to a multipurpose storage phosphor screen, and analyzed using a Cyclone storage phosphor system (Packard).

### Two-sample logo analysis and molecular dynamics simulations

Sequence motif analysis was performed with a Two-Sample logo tool (t-test) [25] using up to a +7,−7 residue window around each modified phospho- Ser/Thr identified. These data were compared with the +7, −7 residue window surrounding Ser/Thr residues randomly extracted from the human proteome obtained from the Swiss Prot database using a homemade script and unix text processing commands. Non-redundant sequences have been randomized using unix command shuff.

*Bona fide* CK2, PLK1, and CK1δ substrates (+7, −7 residue window) were collected from PhosphositePlus database [26] and analysed using Two-Sample Logo vs random Ser/Thr peptides as described above.

Molecular dynamics (MD) simulations of peptide, PLK2, and ATP inserted manually in the active site, was studied using Desmond-Maestro. MD simulations of the minimized complexes (parameterized with OPLS 2005) were performed in order to verify their stability over time; in particular a 70 ns of NPT (1 atm, 300 K) MD simulation was performed.

## Results and Discussion

### Identification of the PLK2 phosphopeptidome

The workflow utilized for the identification of PLK2 peptide substrates is shown in Figure 1. We have generated a peptide library from undifferentiated human neuronal SK-NB-E cells that has been subjected to extensive dephosphorylation by lambda phosphatase. After phosphatase inactivation, the sample has been divided in two equal aliquots. One was incubated with recombinant PLK2 and the other was incubated in the same buffer but without the kinase, as detailed in the methods section. After the reaction, each of the two samples was further split in two identical aliquots. Each aliquot was then separately labeled with the dimethyl labeling reagents, combined (as schematized in Figure 1), subjected to TiO_2_ phosphopeptides enrichment, and finally analyzed by LC-MS/MS. With this approach, we performed a “forward” experiment where the light-labeled sample incubated with PLK2 was mixed with the not phosphorylated medium-labeled sample, and a “reverse” experiment where the medium-labeled sample incubated with PLK2 was mixed with the not phosphorylated light-labeled sample. The stable isotope-based quantification was used to differentiate phosphosites generated by PLK2 from background phosphorylation that could be still present due to an incomplete dephosphorylation reaction. Moreover, for each of the experiments (“forward” and “reverse”) we performed 3 technical replicates, by analyzing the same samples with 3 different fragmentation methods. With this approach we have identified in total 98 unique, PLK2-dependent phosphosites from 89 proteins (Table S1, supplementary material). These phosphopeptides were divided in two categories: the first comprises all phosphopeptides quantified both in the “forward” and in the “reverse” experiment. The reported PLK2-treated/control ratios were calculated as the average value obtained from the technical replicates of each experiment (class 1 phosphopeptides). The second category comprises phosphopeptides that were identified in only one of the experiments (class 2 phosphopeptides) and whose quantification was calculated as the average value obtained from the technical replicates, either in the “forward” or in the “reverse” experiment. All data regarding peptide identifications (protein accession number, peptide sequence, modifications, quantification values, Mascot scores, PEP values, q-values, chromatographic- and MS-relevant information) are reported in Tables S2 and S3, supplementary material.

**Figure 2:**
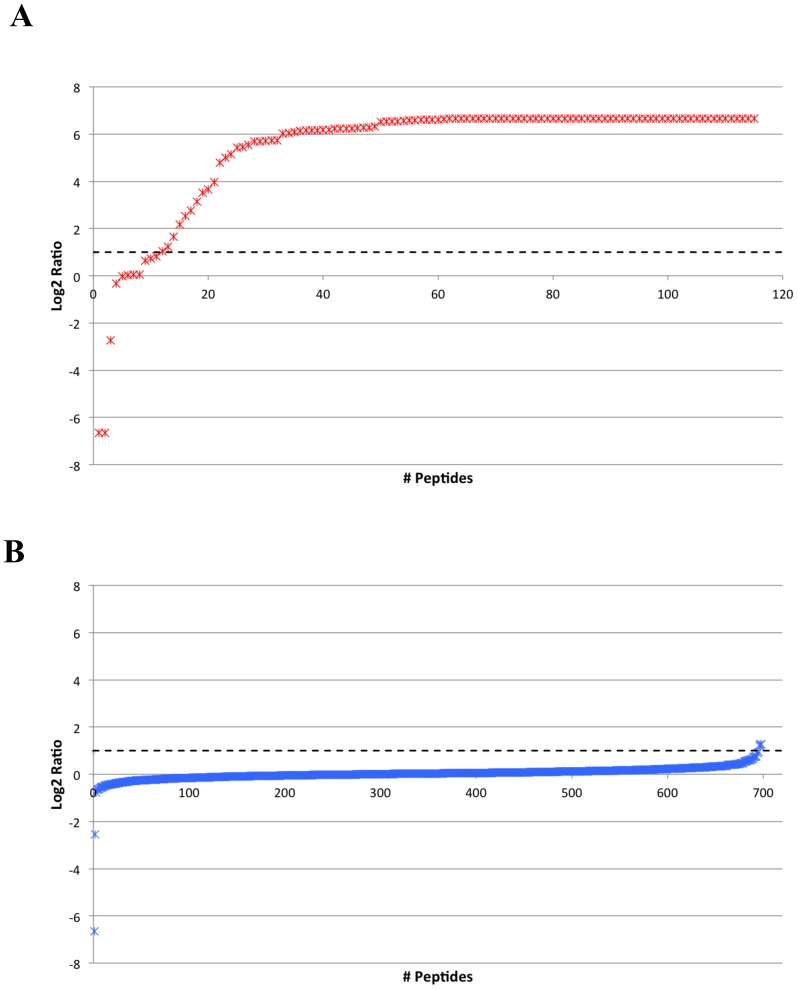
Logarithmic distribution of quantification values. A. Distribution of Log2 ratios relative to all phosphopeptides identified in this study. B. Distribution of Log2 ratios relative to all non-phosphopeptides.

Figure 2 shows the logarithmic distribution of dimethyl label ratios for phosphorylated and non-phosphorylated peptides. In particular, panel A shows the distribution of Log2 ratios relative to phosphorylated peptides, where it is evident that, except for few cases, the very large majority of identified phosphopeptides is present almost exclusively in the sample treated with recombinant PLK2 (the maximum ratio was set at 100, as specified in the methods section). To assess a threshold above which we could consider the fold change as significant, we plotted Log2 ratios for all quantified non-phosphorylated peptides (panel B). As it is possible to see, the Log2 ratio for these peptides never exceeds the value of 1 (dashed line), equivalent to a PLK2-treated/control of 2. Hence this was chosen as the threshold above which the differences between PLK2-treated samples and untreated samples were considered as significant.

### Phosphosites primary structure analysis

The identification of a relatively large number of peptides phosphorylated by PLK2 *in vitro* allowed us to perform a primary structure analysis to define the kinase consensus sequence. Primary structure strongly contributes to the process of substrate recognition, making the determination of the consensus sequence a primary aim for the characterization of a protein kinase. However, it should be borne in mind that other factors may influence the kinase specificity such as tertiary and quaternary structures, and conditions that favor substrate recruitment (for example docking sites not involving the catalytic domain, or the presence of scaffolding and adaptor proteins). Therefore the conformity of a specific substrate to the consensus sequence may be variable [27], [28].

The Two-sample logo is here utilized to obtain a detailed analysis of positive and negative selection of individual residues at given positions around the target site [25]. More in details, this logo provides a graphical representation of the differences between two sets of sequence alignment, i.e. sequences surrounding identified phosphorylated Ser/Thr vs sequences randomly selected from human proteome surrounding Ser/Thr: the upper section displays residues over-represented at a given position in the identified phosphosites as compared to the random one; the lower section displays residues under-represented at a given position in the identified phosphosites.

**Figure 3:**
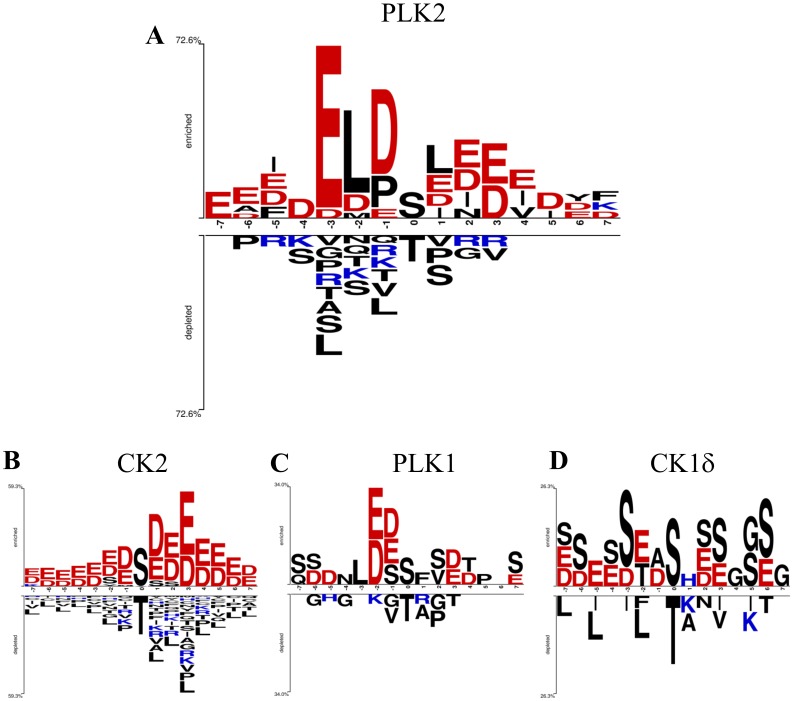
Two-sample logo analysis of phosphosites generated by individual kinases vs. random S/T proteome. PLK2 phosphopeptides identified in this paper (A) or *bona fide* CK2 (B), PLK1 (C), and CK1δ (D) substrates collected from PhosphositePlus database, have been analyzed as described in Materials and Methods.

Several considerations can be made observing the Two-sample logo of Figure 3A. Foremost this analysis confirms the acidophilic nature of PLK2 (initially observed by Johnson *et al.* [29]), showing an enrichment of acidic residues in all positions considered. Positions upstream from the site of phosphorylation (in particular from −3 to −1) display a higher selection consistent with previous observations that the specific determinants of PLK2 are mostly located on the N-terminal side of the target residue [13], [20], [30]. Moreover the main determinants in PLK2 target selection here identified correlate well with previous observations [13], [20], [30].

Particularly remarkable is the striking overrepresentation of glutamic acid at position n-3, present at a frequency of 75% in the identified phosphosites, followed by leucine at −2 and aspartic acid at −1 present at 62,5% and 59%, respectively.

The Two-sample logo generated on PLK2-phosphorylated peptides can be compared with those generated using *bona fide* substrates of the most common acidophilic kinases, i.e. CK2α, CK1δ, and PLK1 (Figure 3). This comparative analysis shows that the four acidophilic kinases present a distinct substrate specificity. Even if all these kinases show an acidophilic nature in substrate recognition, the main acidic determinants are indeed observed at different positions: −3 and −1 for PLK2, +1 and +3 for CK2α, −2 and −1 for PLK1 (Figure 3). In the case of CK1δ the picture is less clear, revealing, besides a “background” of acidic residues at all nearby positions (especially upstream), the recurrent selection of seryl residues reflecting the canonical primed consensus of CK1 (pS-X-X-S) [31]. It is noteworthy that the two-sample logo of PLK2 displays a significant preference for an acidic residue at +3 position that corresponds to the major acidic determinant for CK2 phosphorylation. Moreover about 10% of the identified PLK2 phosphosites presents the strict CK2 consensus sequence s/t[DE]x[DE], thus suggesting a partial target overlap between these two kinases.

**Figure 4:**
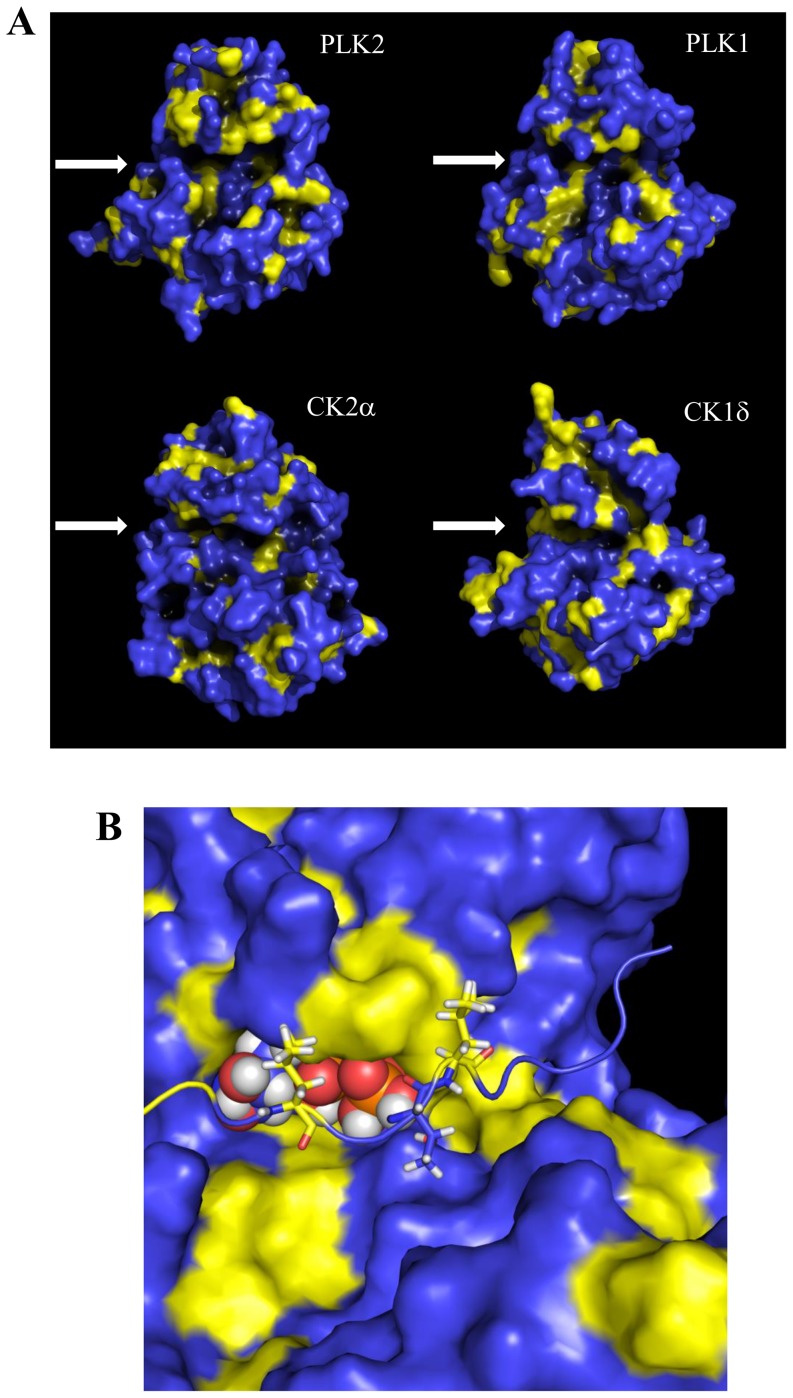
*In silico* analysis of substrate binding zone of PLK2. A. Hydrophobic surface calculation of acidophilic kinases PLK2, PLK1, CK2α, CK1δ. In yellow the hydrophobic areas. Kinase active sites have been indicated by an arrow. B. Interaction between PLK2 and the phosphopeptide EAIAELDtLNEESYK (P31946). −3 and +1 leucine residues are shown in yellow, threonine in blue. ATP is shown in spheres.

Of special interest is the enrichment in hydrophobic residues close to the PLK2 target residue, at −2 (the above-mentioned leucine) and at +1 position. The preference for hydrophobic residues is uncommon among acidophilic kinases even if this feature is shared with PLK1 [29]. Therefore we decided to further investigate this aspect. To provide a structural basis for this enrichment in hydrophobic residues at −2 and +1 position, an *in silico* analysis of the substrate binding zone of PLK2 was performed. Analyzing the hydrophobic amino acid distribution of PLK2 (Figure 4A) it is possible to observe the presence of hydrophobic regions in the active site (yellow areas). These hydrophobic regions, albeit less pronounced, are also present in the active site of PLK1 that also displays a preference for hydrophobic residues at −3 and +1 position (Figure 3C). By sharp contrast, these two hydrophobic regions are absent in the acidophilic kinases CK2 and CK1δ active sites (Figure 4A) consistent with the aminoacid preference observed in Figure 3.

To better analyze this interaction a series of protein-protein docking experiments between PLK2 and one of the phosphopeptides identified in this study EAIAELDtLNEESYK (P31946) were performed. From this analysis it is possible to observe that these hydrophobic regions are responsible for the interaction with the leucine at position −2 and with the hydrophobic residue at position +1, thus further supporting this peculiar feature of PLK2 specificity (Figure 4B).

### Potential novel substrates of PLK2

**Figure 5:**
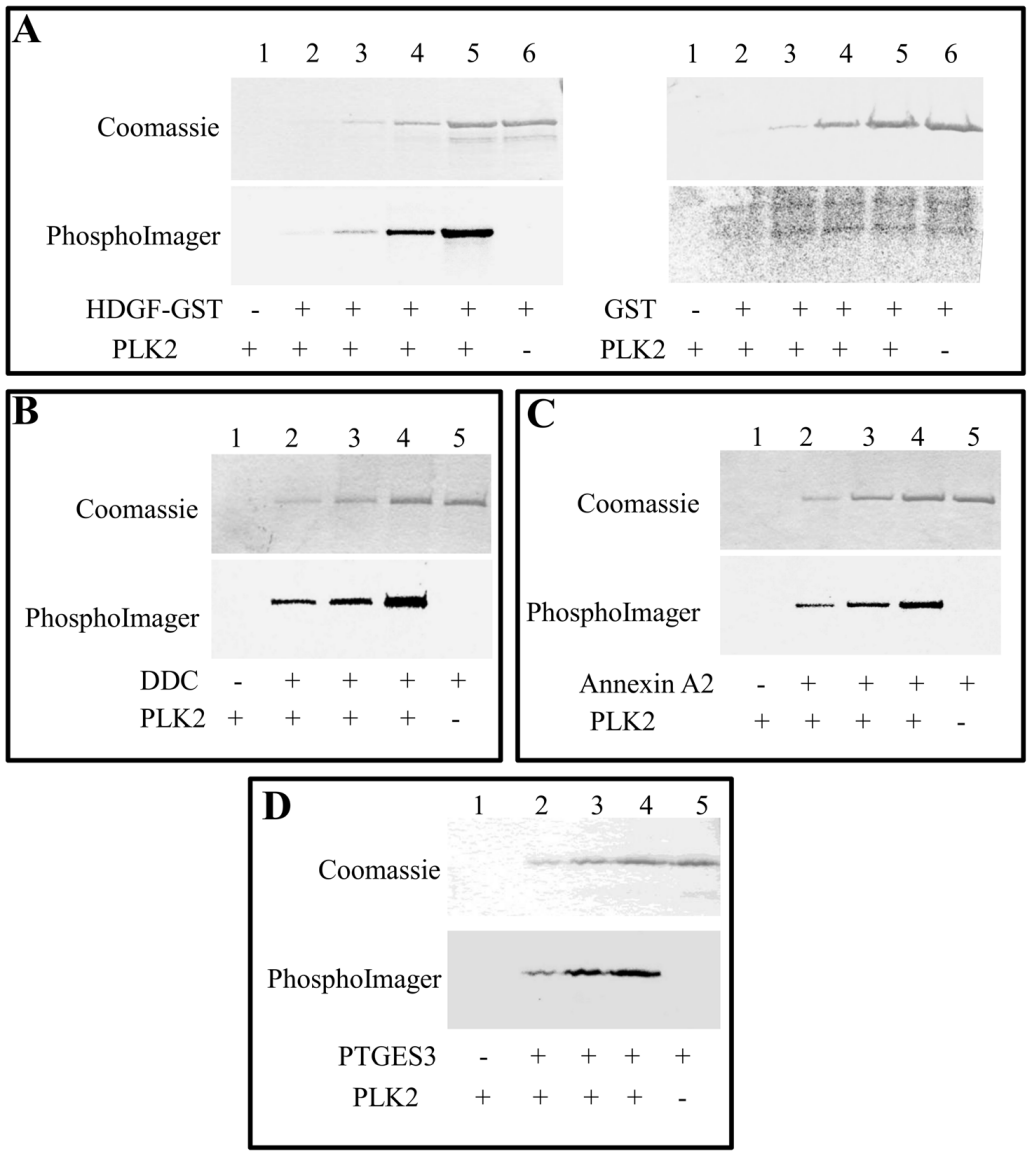
In vitro phosphorylation of recombinant proteins by PLK2. A. Increasing amounts of recombinant GST-HDGF (lane 2, 50 ng; lane 3, 100 ng; lane 4 250 ng; lane 5 and 6, 500 ng) were incubated in radioactive mixture in presence (lanes 1–5) of absence (lane 6) of PLK2 recombinant kinase as described in Materials and Methods. B–D Increasing amounts of purified proteins (lane 2, 100 ng; lane 3, 250 ng; lane 4 and 5, 500 ng) were incubated in radioactive mixture in presence (lanes 1–4) of absence (lane 5) of PLK2 recombinant kinase as described in Materials and Methods. Samples were loaded on SDS-PAGE, stained with colloidal coomassie and ^33^P incorporation was analyzed by PhopshorImager. A- Hepatoma-derived growth factor. B- Aromatic L-amino acid decarboxylase (Dopa decarboxylase). C- Annexin A2. D- Prostaglandin E synthase 3 (PTGES3).

Having used tryptic peptides derived from undifferentiated human neuronal cells as PLK2 *in vitro* substrates, the identified phosphopeptides may help to predict putative PLK2 substrates *in vivo*. Although a residue phosphorylated within a peptide not necessarily undergoes phosphorylation in the full length protein, some observations suggest a good correlation between the phosphopeptidome and the phosphoproteome: two of the substrates identified in fact, i.e. 14-3-3 epsilon and endoplasmin, have been previously identified as *in vitro* protein substrates [20], moreover we have also randomly selected from this list four proteins that have been subjected to *in vitro* phosphorylation by PLK2. All four proteins, GST-HDGF but not GST alone, Annexin A2, Aromatic L-amino acid decarboxylase (Dopa decarboxylase), and Prostaglandin E Synthase 3, were efficiently phosphorylated *in vitro* by PLK2 recombinant kinase (Figure 5). Two of these substrates were further analysed to confirm that the site phosphorylated within the intact proteins corresponds to that identified in the phosphopeptidome (see Figure S1).

These observations strongly support the idea that the newly identified phosphosites are physiologically relevant and can provide new insights into the role of PLK2 in cells. In this connection, we have checked if the phosphosites here identified are already annotated in PhosphositePlus database (www.phosphosite.org) [26]. About 40% of the phosphosites identified in this study have been reported as phosphorylated in cell/*in vivo*. The list of these proteins is shown in Table 1, together with the indication of the phosphosites and, if known, of the kinase/s responsible for their generation. About 90% of these phosphosites are “orphan”, meaning that the kinase/s responsible for their generation are not known.

**Table 1:** List of phosphosites identified in this study as PLK2 substrates that are present in Phosphosite database.

Acc. Number	Name	P-Site	Kinase
P31946	14-3-3 protein beta/alpha	T207	No
P62258	14-3-3 protein epsilon	T208	PLK2/PLK3
P63104	14-3-3 protein zeta/delta	T205	No
Q02952	A-kinase anchor protein 12	S381	No
Q9H4A4	Aminopeptidase B	T408	No
Q9Y2×7	ARF GTPase-activating protein GIT1	S643	No
Q07021	Complement component 1 Q subcomponent-binding protein	S201	No
Q14566	DNA replication licensing factor MCM6	S762	No
P55265	Double-stranded RNA-specific adenosine deaminase	S481	No
P24534	Elongation factor 1-beta	S95	No
P14625;P08238	Endoplasmin/Heat shock protein HSP 90-beta	S106/S45	No
Q9H501	ESF1 homolog	S663	No
P55884	Eukaryotic translation initiation factor 3 subunit B	S152	No
P56537	Eukaryotic translation initiation factor 6	S175	CK1δ
P35269	General transcription factor IIF subunit 1	S218	No
O60763	General vesicular transport factor p115	S942	CK2/GCK
P08238	Heat shock protein HSP 90-beta	S365	No
P51858	Hepatoma-derived growth factor	T225	No
P31943/P55795	hnRNA H1/hnRNP H2	S63	No
P17096	High mobility group protein HMG-I/HMG-Y	S99	No
P46821	Microtubule-associated protein 1B	S1156	No
Q14978	Nucleolar and coiled-body phosphoprotein 1	S637	No
Q9NR30	Nucleolar RNA helicase 2	S84	No
Q9NR30	Nucleolar RNA helicase 2	S121	No
P19338	Nucleolin	S28	No
P09874	Poly [ADP-ribose] polymerase 1	S785	No
Q99623	Prohibitin-2	S119	No
Q15185	Prostaglandin E synthase 3	S113	CK2
Q15084	Protein disulfide-isomerase A6	S428	CK2
P13521	Secretogranin-2	S104	No
Q13813	Spectrin alpha chain, non-erythrocytic 1	S391	No
Q96I25	Splicing factor 45	T224	No
Q13428	Treacle protein	S270	No
P40939	Trifunctional enzyme subunit alpha, mitochondrial	S669	No
P60174	Triosephosphate isomerase	S260	No
G3V1U9;P68363	Tubulin alpha-1A chain/Tubulin alpha-1B chain	S48	No
Q9BVA1;Q13509;P07437	Tubulin beta-2B/Tubulin beta-3/Tubulin beta	T72	No
P68371;P07437	Tubulin beta-4B chain/Tubulin beta chain	S126	No
P15374	Ubiquitin carboxyl-terminal hydrolase isozyme L3	S161	No
Q15942	Zyxin	S150	No

**Figure 6:**
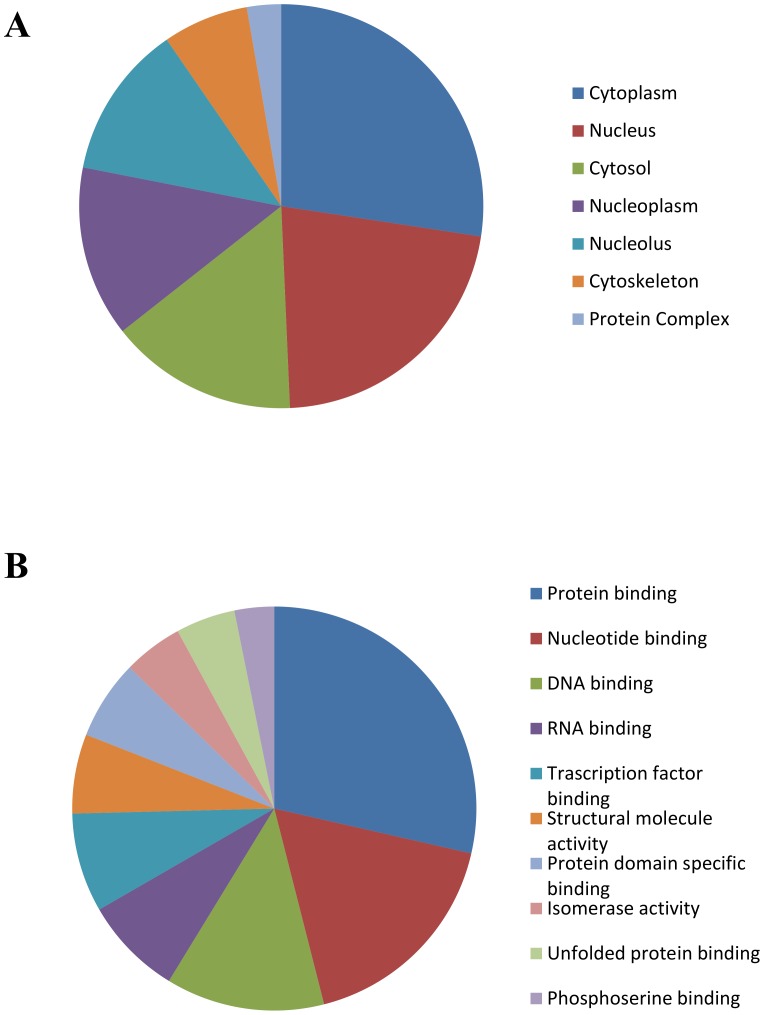
Putative PLK2-substrate localization (A) and functional (B) analysis. Subcellular localization (A) and functional analysis (B) for each protein have been assigned using GeneCoDis3 webserver [35], [36].

Figure 6 shows the analysis of subcellular localization (A) and molecular functions (B) of putative PLK2 substrates identified in this study. Identified proteins localize both in cytoplasmic and nuclear compartments and participate to several processes where the involvement of PLK2 kinase has not been described yet. As mentioned above the number of *bona fide* PLK2 substrates identified so far is low and includes not only cytosolic proteins, but also plasma membrane [32] and nuclear [33] substrates. The localization of PLK2 at centrosomes where it regulates centriole duplication, has been deeply investigated [4]. However PLK2 has been identified also in different subcellular compartments, such as cytoplasm, nucleus (PLK2 contains a nuclear localization signal [34]), and membranes in HEK 293T cells [12], while in primary hippocampal neurons PLK2 shows primarily a nuclear localization [12]. Co-localization between the kinase and its putative substrates suggests unanticipated regulatory roles for PLK2 in nuclear functions.

Finally, given the known role of PLK2 in synaptic remodeling, it would be interesting to extend the analysis also to a model of differentiated neuronal cells, such as human cortex or primary neuron cultures. This approach could reveal substrates of PLK2 that are only expressed at the synapse and that were not identified in the present study. This will increase the panel of putative substrates of PLK2 and, on the other hand, will allow to identify substrates correlated to specific neuronal functions.

## Supporting Information

Figure S1 **Confirmation of PLK2 phosphorylation sites in intact proteins.** A. 200 ng (lane 1) or 400 ng (lane 3) of GST-HDGF wild type and 200 ng (lane 2) or 400 ng (lane 4) of GST-HDGF T225A were incubated for 10 minutes in the radioactive mixture as described in the Material and Methods section in presence of PLK2 (left panel) or CK2 (right panel), loaded in SDS-PAGE gel, coomassie stained and analyzed by PhosphorImager. B. Prostaglandin E Synthase 3 (400 ng) was phosphorylated by recombinant PLK2 as in Figure 5, loaded in SDS-PAGE gel, coomassie stained, and trypsin digested. Phosphopeptides were enriched and identified as described in Material and Methods. The annotated MS/MS spectrum relative to the phosphopeptide DWEDDpSDEDMSNFDR is displayed together with all relevant information regarding peptide identification.
(TIF)

Table S1 **List of phosphopeptides specifically phosphorylated by PLK2.** The Table lists all phosphopeptides identified in this study with a PLK2-treated/control ratio above 2. The ratios were obtained as the average values from all technical replicates. Class 1 phosphopeptides were quantified both in the “forward” and in the “reverse” experiment, while class 2 phosphopeptides were quantified only in one of the experiments. Stretches of sequences in brackets indicate that the same phosphosite was found in peptides with different number of missed-cleavages.
(XLSX)

Table S2 **Relevant information relative to the peptides identified in the “forward” experiment.** The table lists the sequences of all identified peptides, together with protein accession numbers, modifications, quantification values, Mascot scores, PEP values, q-values, chromatographic- and MS-relevant information.
(XLSX)

Table S3 **Relevant information relative to the peptides identified in the “reverse” experiment.** The table lists the sequences of all identified peptides, together with protein accession numbers, modifications, quantification values, Mascot scores, PEP values, q-values, chromatographic- and MS-relevant information.
(XLSX)
