# Correction: Identification of the PLK2-Dependent Phosphopeptidome by Quantitative Proteomics

**DOI:** 10.1371/journal.pone.0114202

**Published:** 2014-11-18

**Authors:** The PLOS ONE Staff

## Notice of Republication

This article was republished on October 30, 2014, due to an error in the title. The publisher apologizes for this error. The citation has been updated to match the corrected title.
