# Supplementary figures and images for: Identification of the PLK2-Dependent Phosphopeptidome by Quantitative Proteomics
Source: PLoS One. 2014 Oct 22;9(10):e111018. doi: 10.1371/journal.pone.0111018 (PMC4206460; doi:10.1371/journal.pone.0111018)

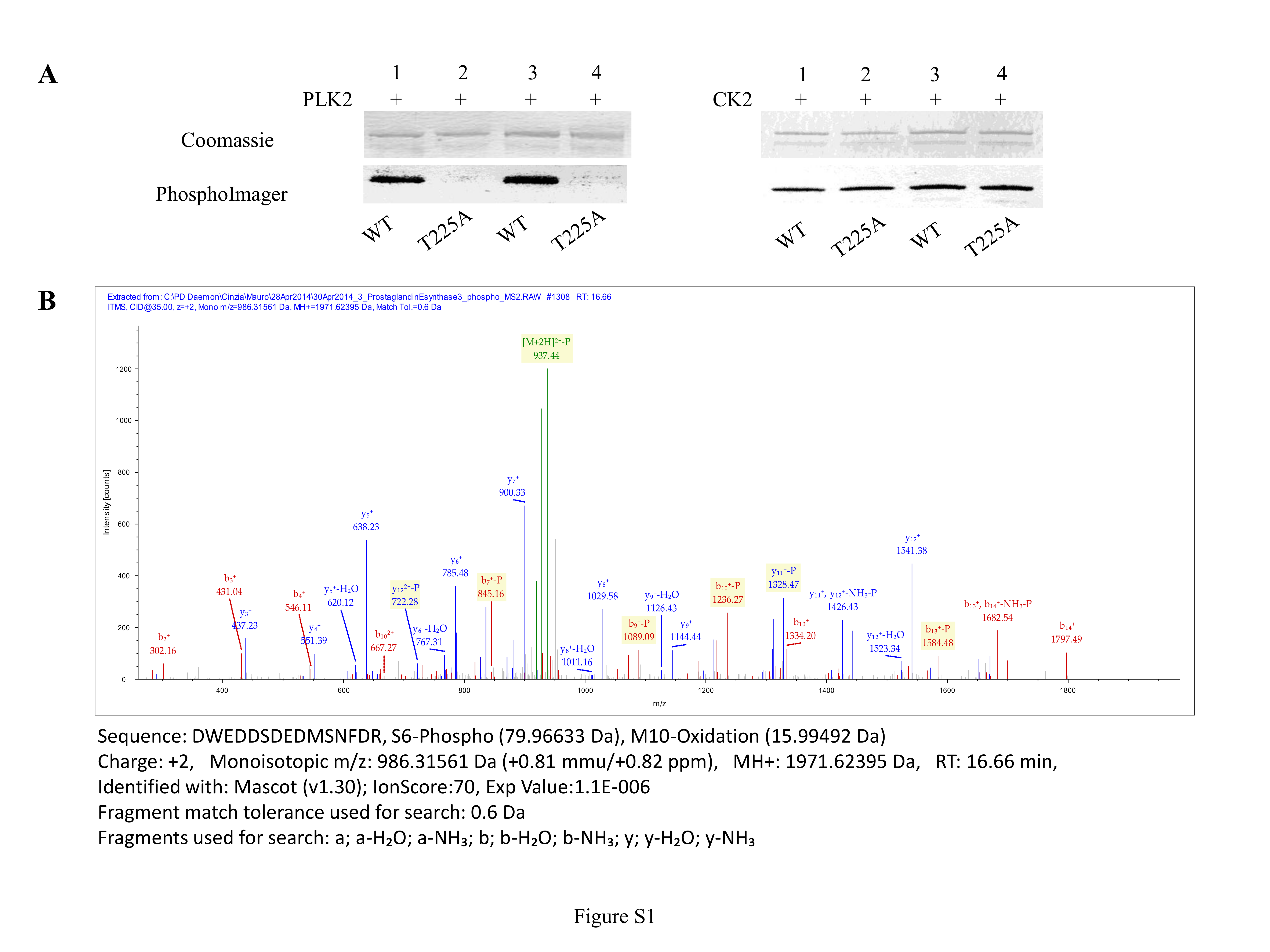

Supplement: Figure S1 — Confirmation of PLK2 phosphorylation sites in intact proteins. A. 200 ng (lane 1) or 400 ng (lane 3) of GST-HDGF wild type and 200 ng (lane 2) or 400 ng (lane 4) of GST-HDGF T225A were incubated for 10 minutes in the radioactive mixture as described in the Material and Methods section in presence of PLK2 (left panel) or CK2 (right panel), loaded in SDS-PAGE gel, coomassie stained and analyzed by PhosphorImager. B. Prostaglandin E Synthase 3 (400 ng) was phosphorylated by recombinant PLK2 as in Figure 5, loaded in SDS-PAGE gel, coomassie stained, and trypsin digested. Phosphopeptides were enriched and identified as described in Material and Methods. The annotated MS/MS spectrum relative to the phosphopeptide DWEDDpSDEDMSNFDR is displayed together with all relevant information regarding peptide identification. (TIF) [file pone.0111018.s001.tif]
